# Juvenile Dermatomyositis in Afro-Caribbean children: a cohort study in the French West Indies

**DOI:** 10.1186/s12969-023-00904-w

**Published:** 2023-10-07

**Authors:** Arthur Felix, Frederique Delion, Fabienne Louis-Sidney, Lindsay Osei, Aurélie Armougon, Remi Bellance, Moustapha Dramé, Christophe Deligny, Benoit Suzon, Yves Hatchuel

**Affiliations:** 1 Department of General Pediatrics, Competence Centre for Rheumatic, Autoimmune and Systemic diseases in children (RAISE) Antilles-Guyane, EpiCliV Research Unit, University of the French West Indies, Martinique University Hospital, MFME. CHU de la Martinique La Meynard, 97261 Fort-de France, France; 2 Department of Pediatrics, Guadeloupe University Hospital, Pointe-À-Pitre, France; 3 Department of Rheumatology, Martinique University Hospital, Fort-de-France, France; 4 Department of Pediatrics, Andrée Rosemon Hospital, Cayenne, France; 5 Neurological and Neuromuscular Rare Disorders Department, CERCA, University Hospital Center of Martinique, Martinique, France; 6 Department of Clinical Research and Innovation, Martinique University Hospital, Fort-de-France, France; 7 Department of Internal Medicine, Martinique University Hospital, Fort-de-France, France

**Keywords:** Juvenile dermatomyositis, Auto-immune myositis, Afro-Caribbean children, African descent children

## Abstract

**Introduction:**

The epidemiology of Juvenile Dermatomyositis (JDM) in non-Caucasian population is poorly described. We performed a study of patients followed up in the French West Indies for JDM. We aimed to describe clinical and biological specificities during childhood.

**Methods:**

Retrospective study covering the period from Januarys 2000–2023. Listings of patients were obtained from multiple sources, namely computerized hospital archives, registry of referent pediatricians and adult specialists in internal medicine and the French National Registry for rare diseases. JDM and organ involvement were defined according to the international ILAR criteria.

**Results:**

Twenty-one patients were included over a 23 year-period. Median age at onset was 8.1 years (Range: 2.5—13.9) with a median follow up of 8 years (Range: 2—19). Two-thirds (14/21) had dysphagia at onset and 33% had respiratory involvement. Thirteen had specific autoantibodies (58%), most frequently anti-Mi-2. The median number of flares during childhood was three (1—9). During childhood, 76% had calcinosis lesions. Clinical evolution seemed to be more aggressive for boys than girls (respectively 4.2 versus 2.2 flares (*p* = 0.04) and 50% vs 18% needing more than one background therapy, *p* = 0.03).

**Conclusion:**

This retrospective study is the largest cohort of pediatric patients of Afro-Caribbean and Black African descent treated for JDM in a high-income health system, and the first to describe the incidence and immunological profile in a population of African descent. They had higher rate of calcinosis and similar respiratory involvement. Overall outcomes during childhood were similar to North America and European countries.

**Supplementary Information:**

The online version contains supplementary material available at 10.1186/s12969-023-00904-w.

## Key messages

1. First cohort of pediatric patients of Afro-Caribbean origin with Juvenile Dermatomyositis

2. Higher rate of calcinosis, same rate of respiratory involvement

3. Incidence and overall prognosis similar to children in North America and European countries

**Supplementary Information:**

The online version contains supplementary material available at 10.1186/s12969-023-00904-w.

## Introduction

In populations of African descent, the clinical presentation and epidemiology of auto-immune and auto-inflammatory diseases is quite particular [1, 2]. The epidemiology and clinical features of Juvenile Dermatomyositis (JDM) in populations of African descent has not been well described heretofore, except in one small series from South Africa [3]. It has never been described in an Afro-Caribbean population. The French West Indies (FWI, i.e. Martinique, Guadeloupe, and French Guyana) have a combined population of approximately 330,000 children aged under 18 years [4]. The healthcare system is free and universal, with two university hospitals and reference centers approved by the French Ministry of Health. Although there are no official ethnicity statistics, a large majority of patients are of black African descent [5] (> 90%). The objectives of this study were to perform a retrospective, descriptive study, reported according to the STROBE methodology [6], of patients from the FWI followed-up for JDM. We aimed to describe clinical, immunological specificities and outcomes during childhood.

## Methods

This was a retrospective study covering the period from January 2000 to January 2023. The methods used to identify patients aimed to cross-reference different sources to ensure exhaustive identification of all patients. In each reference center of the FWI, we searched the local registries of pediatric patients followed-up for JDM by the referring pediatricians. We also extracted lists of patients from the electronic hospital archives and the French Medicalization of Information Systems Program (PMSI), which is a comprehensive national database that contains all hospital discharge records, using the coded diagnosis of JDM (M33, M608, and M609). We also extracted the list of patients recorded in the electronic French national registry for rare diseases (BAMARA), a secure national information system. Subsequently, the lists of patients were analyzed for the relevance of the diagnoses, to check inclusion criteria and to eliminate duplicates. Detailed information on the number of patients identified according to the databases and the number of duplicates is summarized in Supplementary Fig. 1. The disease studied was JDM according to international ILAR criteria [7]. Respiratory involvement was defined as Interstitial lung disease and/or abnormalities of the Pulmonary Function Tests (PFT) such as respiratory restrictive syndrome, non-reversible obstructive syndrome associated with diffusing capacity of carbon monoxide (DLCO) abnormality. Patients with dysphagia or aphagia were systematically evaluated by an ENT specialist. A clinical flare was described as an exacerbation, biologically confirmed by an increase in muscle enzymes, with muscle weakness and/or dysphagia or respiratory involvement requiring a change in background therapy or steroid pulse (> 0.5 mg/kg/day). For muscular forms without other organ involvement, the local treatment protocol consisted of intravenous corticosteroid therapy followed by oral corticosteroid therapy. If corticosteroids could not be discontinued after 3 months or if there was a relapse, patients were given a corticosteroid-sparing agent, methotrexate (MTX) as the first-line treatment or Mycophenolate Mofetil (MMF) if MTX failed. In the case of severe gastrointestinal or ENT involvement, either polyvalent immunoglobulins or plasma exchange were added. Clinical forms refractory to conventional treatment benefited from Janus Kinas inhibitors. Patients were included if they were born in the FWI. Differences in percentages were tested with the chi square test or Fisher’s exact test for expected frequencies < 5, using STATA software. A *p*-value < 0.05 was considered statistically significant. The Institutional Review Board of the University Hospital of Martinique approved the study under the number 2021/116.

## Results

Twenty-one patients suffering from JDM were identified over the 23-year study period. The incidence of JDM was 0.3 patients per year per 100,000 children. Their clinical and immunological features are shown in Table 1. The flowchart is shown in Supplementary Fig. 1. Overall, all patients were found across at least 2 different database sources (number of duplicates 100%). The median follow up was 8 years (range: 2—19). The median time to diagnosis was 2 months (range: 0 – 11 months). The clinical presentation was muscle weakness and pain with motor deficit (100%), Gottron papules or Gottron sign (95%), fever (81%), inflammatory arthralgia or arthritis (67%). The average Manual muscle test (MMT) at onset was 50/80 (Range: 29–70) and average Childhood Myositis Assessment Scale (CMAS) was 27/52 (Range: 1–48). Two thirds (14/21) of our patients had dysphagia at onset. Five patients had respiratory involvement at onset (24%) and two patients developed pulmonary involvement later during childhood. Overall, seven children had respiratory involvement in our cohort (33%); this was unrelated to age at onset or sex. No patient with anti-MDA5 antibody had pulmonary involvement in our cohort. Five patients had digestive involvement with severe constipation. We found a viral infectious trigger in the first flare (positive nasal or blood PCR) in 15/21 patients (71%). The viruses identified were human influenza virus (27%), human parvovirus B19 (20%), respiratory syncytial virus (20%)SARS-CoV-2 (13%), parainfluenza virus (13%), Epstein Barr virus (7%). Seventeen patients (81%) had biopsy confirming the diagnosis, 14/21 had body MRI showing diffuse inflammatory myositis (67%), 13 had proven specific myositis autoantibody (58%), most frequently anti-Mi-2 (Table 1). Even when biological tests were repeated over time, no autoantibodies were found in patients who were seronegative at diagnosis.

**Table 1:**
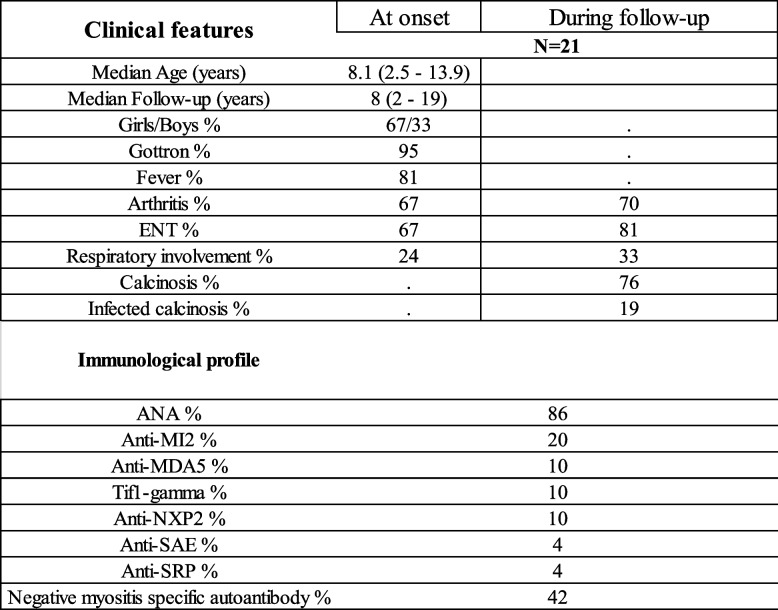
Clinical and biological features of pediatric patients with juvenile dermatomyositis

*ENT* ear, nose, and throat; *ANA* Antinuclear Antibodyt

**Fig. 1:**
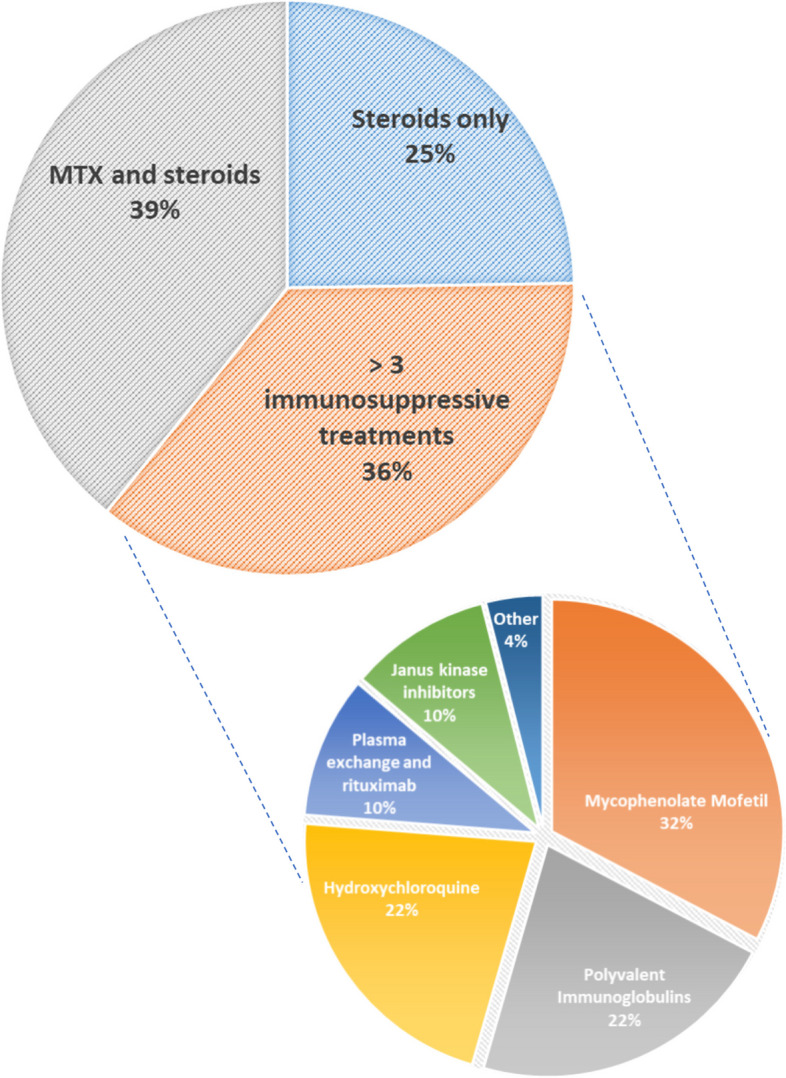
Background therapy throughout childhood for Juvenile dermatomyositis. MTX, Methotrexate. Initial treatment of a flare was based on high-dose steroid therapy gradually tapered and weaned over 3–6 months. Corticosteroid-sparing treatment was administered in the event of corticosteroid dependence or relapse

The average and median number of flares during childhood was three per patient (1—9). Thirteen patients were withdrawn from steroids during childhood or at the date of last news (62%). Seven patients (33%) needed plasma exchange and/or polyvalent immunoglobulins at onset for severe forms, mostly associated with severe dysphagia and digestive involvement. Two patients diagnosed after 2018 received Janus kinase inhibitors for respiratory involvement (Fig. 1). During childhood, 76% developed calcinosis lesions (16/21), which resulted in four abscess complications requiring surgery.

There were three profiles of disease progression during childhood. Six patients only had 1–2 flares without dysphagia, digestive or respiratory involvement; the flares were rapidly regressive with treatment and needed only steroids and no other treatment (25%). Most of these patients had anti-Mi2 antibodies (4/6). Eight patients had 2–3 flares during childhood and required initiation of corticosteroid-sparing treatment (mostly MTX) (39%). Most of these had dysphagiabut not respiratory involvement at onset (7/8), were seronegative at onset and during follow-up. Seven patients had multiple (> 3) and severe relapses (5–9) and needed 3 to 6 lines of immunosuppressive treatment during childhood (MTX, MMF, hydroxychloroquine, rituximab, plasmapheresis, intravenous immunoglobulin, Janus Kinase inhibitors) (36%). Most of the patients with these severe forms were boys (4/7), and this disease severity was unrelated to age at onset (onset severe forms 7.4 vs 7 years old, *p* = 0.61). These severe phenotypes were associated with Anti-NXP2 antibodies. The Janus Kinase inhibitors were effective to induce remission in these severe forms with respiratory and digestive involvement (Fig. 1).

Clinical progress during childhood seemed to be more aggressive for boys than girls (average number of flare during childhood 4.2 versus 2.2 for girls (*p* = 0.04), 50% vs 18% needed more than one background therapy, *p* = 0.03). There were no chronic respiratory failure or ENT sequelae, and no death recorded in our cohort. No patient had malignancy during follow-up.

## Discussion

This retrospective study from the FWI over a period of 23 years identified a cohort of 21 patients suffering from JDM. This is the largest cohort of pediatric patients of Afro-Caribbean and Black African descent with JDM.

One of the strengths of this study is its multicenter nature, with the participation of all the referring pediatricians in the FWI. Our methodology enabled us to identify patients and their therapies by referring to the national registry for rare diseases, as well the registries of local clinicians and by exploring computerized hospital archives. This led to exhaustive identification of patients, and we cross-checked data from multiple sources to minimize the loss of patients and data. Thus, the incidence in our study may have been underestimated, because of the bias of retrospective studies in rare diseases covering a long period. There is also potential for recruitment bias in our study, because our methodology mostly identifies patients who required hospitalization. Nevertheless, most patients with JDM have a short initial hospitalization at disease onset.

The clinical presentation of patients was generally clear and flagrant, with florid symptoms compared to European and Latin-American studies, where 40% of insidious forms are described [8, 9]. Overall, the outcomes of Afro-Caribbean children followed-up and treated in the FWI for JDM was comparable to North America and European countries. This florid symptomatology at diagnosis with a similar overall prognosis has previously been described as a characteristic of our population in another connective tissue disease namely juvenile systemic lupus [10]. In studies from North America or Africa, African descent seems to be associated with worse prognosis in JDM [3, 11, 12]. However, it remains unclear whether this association is due to genetic differences, environmental exposure, access to care, treatment adherence, or other, unknown related factors. The French healthcare system is universal and free, therefore, the bias related to socioeconomic status and access to healthcare should be less significant in our cohort.

Calcinosis occurs in 40% of patients with JDM, although current reported prevalence ranges from 10 to 70% [13, 14]. In our cohort, we found a high rate of calcinosis complicating JDM (76%). This ethnic susceptibility, with rates up to 70% for populations of African descent has previously been described [11, 15]. These high rates of calcinosis in African descendants suggest a genetic component in the pathogenesis of calcinosis.

The incidence of lung involvement during childhood (33%) was comparable to pediatric series from North America and European countries [16]. However, it was higher than usually described for populations of African descent, considered less at risk [16]. Sixty-two percent of patients had positive specific antibodies, even if the biological assays were repeated over time. This frequency was comparable to larger cohorts [17]. Our study is the first to show the immunological profile of JDM in children of African descent. The pattern of clinical severity and more aggressive treatment to achieve clinical remission has already been described in NXP2 associated JDM [18].

Our study shows an incidence of JDM in our Afro-Caribbean children at a rate of 0.3 patients per year per 100,000 children. This incidence is similar to North America and European countries [19]. In contrast, the incidence of another connective tissue disease (lupus) is much more important in our Afro-Caribbean population in the FWI [10].

## Conclusions

To the best of our knowledge, our cohort is the largest focusing on children of Afro-Caribbean origin and treated for JDM in a high-income health system and the first to describe the incidence and immunological profile in a population of children of African descent. It describes their clinical specificities, such as a higher rate of calcinosis and similar rates of respiratory involvement. Overall prognosis in terms of mortality and chronic organ damage during childhood was similar to North America and European countries.

### Supplementary Information

**Additional file 1:** **Supplementary Figure 1. **Flowchart of the study population.

## Data Availability

All data generated or analyzed during this study are included in this published article and tables.

## Abbreviations

JDM: Juvenile Dermatomyositis
FWI: French West Indies
ENT: Ear, nose, and throat
PMSI: French Medicalization of Information Systems Program
PFT: Pulmonary Function Tests
DLCO: Diffusing capacity of carbon monoxide
MMT: Manual muscle test
CMAS: Childhood Myositis Assessment Scale
PCR: Polymerase chain reaction
MTX: Methotrexate
MMF: Mycophenolate Mofetil

## References

[1] Chipeta J, Njobvu P, Wa-Somwe S, Chintu C, McGill PE, Bucala R (2013). Clinical patterns of juvenile idiopathic arthritis in Zambia. Pediatr Rheumatol Online J.

[2] Olaosebikan BH, Adelowo OO, Animashaun BA, Akintayo RO (2017). Spectrum of paediatric rheumatic diseases in Nigeria. Pediatr Rheumatol Online J.

[3] Okong’o LO, Esser M, Wilmshurst J, Scott C (2016). Characteristics and outcome of children with juvenile dermatomyositis in Cape Town: a cross-sectional study. Pediatr Rheumatol..

[4] Felix A, Delion F, Suzon B (2022). Systemic juvenile idiopathic arthritis in French Afro-Caribbean children, a retrospective cohort study. Pediatr Rheumatol.

[5] Levinson: Ethnic groups worldwide: a ready reference... - Google Scholar. Accessed May 30, 2022. https://scholar.google.com/scholar_lookup?hl=en&publication_year=1998&pages=350-350&author=D+Levinson&title=Ethnic+Groups+Worldwide%3A+a+ready+reference+group+handbook.

[6] von Elm E, Altman DG, Egger M (2007). The Strengthening the Reporting of Observational Studies in Epidemiology (STROBE) statement: guidelines for reporting observational studies. Lancet Lond Engl.

[7] Lundberg IE, Tjärnlund A, Bottai M (2017). EULAR/ACR Classification criteria for adult and juvenile idiopathic inflammatory myopathies and their major subgroups. Ann Rheum Dis.

[8] Guseinova D, Consolaro A, Trail L (2011). Comparison of clinical features and drug therapies among European and Latin American patients with juvenile dermatomyositis. Clin Exp Rheumatol.

[9] McCann LJ, Juggins AD, Maillard SM (2006). The Juvenile Dermatomyositis National Registry and Repository (UK and Ireland)–clinical characteristics of children recruited within the first 5 yr. Rheumatol Oxf Engl.

[10] Felix A, Delion F, Suzon B (2022). Systemic lupus of pediatric onset in Afro-Caribbean children: a cohort study in the French West Indies and French Guiana. Pediatr Rheumatol Online J.

[11] Phillippi K, Hoeltzel M, Robinson AB, Kim S (2017). Race, income and disease outcomes in Juvenile dermatomyositis. J Pediatr.

[12] Pachman LM, Hayford JR, Chung A (1998). Juvenile dermatomyositis at diagnosis: clinical characteristics of 79 children. J Rheumatol.

[13] Ravelli A, Trail L, Ferrari C (2010). Long-term outcome and prognostic factors of juvenile dermatomyositis: a multinational, multicenter study of 490 patients. Arthritis Care Res.

[14] Robinson AB, Hoeltzel MF, Wahezi DM (2014). Clinical characteristics of children with juvenile dermatomyositis: the childhood arthritis and rheumatology research alliance registry. Arthritis Care Res.

[15] Faller G, Mistry BJ, Tikly M (2014). Juvenile dermatomyositis in South African children is characterised by frequent dystropic calcification: a cross sectional study. Pediatr Rheumatol Online J.

[16] Sun KY, Fan Y, Wang YX, Zhong YJ, Wang GF (2021). Prevalence of interstitial lung disease in polymyositis and dermatomyositis: a meta-analysis from 2000 to 2020. Semin Arthritis Rheum.

[17] Kobayashi I, Akioka S, Kobayashi N (2020). Clinical practice guidance for juvenile dermatomyositis (JDM) 2018-Update. Mod Rheumatol.

[18] DeWane ME, Waldman R, Lu J (2020). Dermatomyositis: clinical features and pathogenesis. J Am Acad Dermatol.

[19] Mendez EP, Lipton R, Ramsey-Goldman R (2003). US incidence of juvenile dermatomyositis, 1995–1998: results from the National Institute of Arthritis and Musculoskeletal and Skin Diseases Registry. Arthritis Rheum.

