# Supplementary material for: Juvenile Dermatomyositis in Afro-Caribbean children: a cohort study in the French West Indies
Source: Pediatr Rheumatol Online J. 2023 Oct 7;21:113. doi: 10.1186/s12969-023-00904-w (PMC10559605; doi:10.1186/s12969-023-00904-w)
Supplement: Supplementary file 1 — Additional file 1: Supplementary Figure 1. Flowchart of the study population. [file 12969_2023_904_MOESM1_ESM.docx]

**Supplementary figure 1: Flowchart of the study population**

*BAMARA is the French National registry for rare disease.*
